# Development of Application Customization Toolkit (ACT) for 3D Thermal Elastic-Plastic Welding Analysis

**DOI:** 10.3390/ma18010057

**Published:** 2024-12-26

**Authors:** Jaeyong Lee, Dong Hee Park, Juhyeon Park, Do Kyun Kim

**Affiliations:** 1 Department of Naval Architecture, Republic of Korea Naval Academy, 88-1, Changwon 51704, Republic of Korea; 2 Department of Naval Architecture and Ocean Engineering, College of Engineering, Seoul National University, Seoul 08826, Republic of Korea; 3 Ship and Offshore Research Institute, Samsung Heavy Industry Co., Ltd., Geoje 53261, Republic of Korea; 4 Department of Naval Architecture and Ocean Engineering, Pusan National University, Busan 46241, Republic of Korea; 5 Ocean & Shore Technology (OST) Laboratory, Research Institute of Marine Systems Engineering, Seoul National University, Seoul 08826, Republic of Korea

**Keywords:** welding, 3D thermal elastic-plastic welding, welding deformation, residual stress, ultimate compressive strength, T-joint fillet welding

## Abstract

A 3D thermal elastic-plastic welding analysis ACT (Application Customization Toolkit) was developed in ANSYS, making welding analysis more accessible. The welding analysis was performed using a decoupled method, separated into thermal and structural analyses. To validate the results, comparisons were made with previous studies for two types of welding: T-joint fillet welding and butt welding. Subsequently, the residual stress and deformation obtained from the welding analysis were applied as initial imperfections in a compression analysis to evaluate the ultimate compressive strength with conventional compression analysis. This comparison allowed for a more realistic assessment of the effects of deformation and residual stress distribution on the structural behaviours.

## 1. Introduction

Welding is a method of joining two distinct components utilised across various industries. In shipbuilding, welding is widely applied for the fabrication of large steel structures and the connection of reinforcement plates, enabling the construction of massive ship hulls and enhancing structural strength using reinforcement plates. In the case of fusion welding used in shipbuilding, high temperatures are applied to melt the base metal, which is then fused and allowed to solidify upon cooling. During this process, the components undergo thermal expansion and contraction due to heating and cooling, respectively, which can lead to unintended residual stresses and initial distortions. Residual stresses may deduct the structural strength of the hull structure, while initial distortions not only weaken the structure but also alter its dimensions, necessitating additional work and increasing production time [1,2,3].

Therefore, understanding the effects of residual stress and initial distortion caused by welding is crucial. However, due to the features of the ship structure scale, conducting welding experiments on actual components and measuring their impact has been challenging. In an effort to understand the influence of welding through FEM, Deng et al. [4] performed experiments and finite element analysis on T-joint welding, comparing deformations and shrinkage. Perić et al. [5] conducted both experimental and numerical investigations of T-joint fillet welding, examining the residual stress and deformation induced by welding and comparing the results. They also attempted to reduce analysis time, partially by using shell elements. In a subsequent study, Perić et al. [6] conducted a decoupled welding analysis consisting of thermal and structural analyses. During thermal analysis, researchers employed the Element Birth and Death (EBD) method to simulate a moving welding torch while not in structural analysis to save computational costs.

Nevertheless, three-dimensional thermal elastic-plastic analysis still proved to be time-consuming. This led to the development of studies incorporating the concept of inherent strain for welding analysis. Using inherent strain, longitudinal and transverse shrinkage forces and moments were derived from equivalent nodal forces, and welding deformation was determined through elastic analysis by applying these forces as external loads [7]. Ha [8] devised a method for converting inherent strain into thermal boundary conditions to produce deformation equivalent to that generated by applying equivalent loads. Liang & Murakawa [9] aimed to reduce time and costs by measuring coordinates at specific locations through welding experiments, deriving inherent strain via inverse analysis, and conducting elastic FEM. Wang et al. [10] measured deformation in fillet welding and performed elastic FE analysis using inherent strain. However, the inherent strain approach has limitations, being applicable primarily to relatively simple structures.

An analysis toolkit was developed to overcome the challenges between analysis time and accuracy in welding simulations. When developing the toolkit, welding parameters were selected with consideration for the convenience of welding analysis and ensuring continuity with future research. By consolidating various parameters that influence welding, the ease of analysis was enhanced, and the time required for welding simulations was reduced. Experimental results and analysis data from previous studies were compared to validate the analytical method. Furthermore, as previous studies lacked further research after analysing residual stresses, an analysis was conducted on the ultimate limit state assessment of structures considering residual stresses and welding-induced deformations. This included examining the practical effects of residual stresses on structural integrity.

## 2. Methodology

In previous research that conducted parametric studies of welding analysis, welding arc current [11,12,13,14,15,16,17], welding order [14], and weld temperature [18] were usually used.

**Figure 1:**
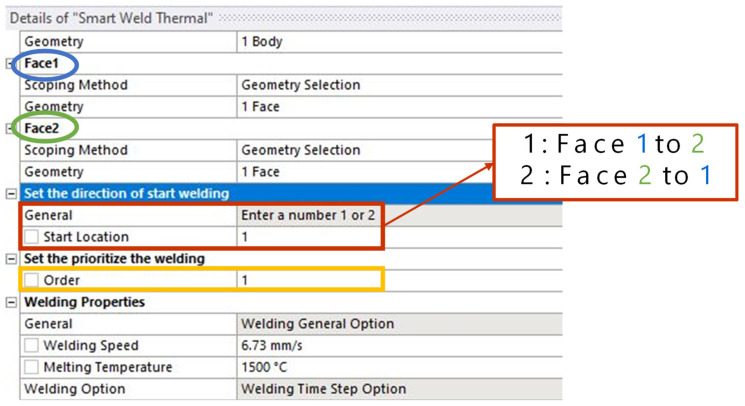
Options of welding direction and order.

**Figure 2:**
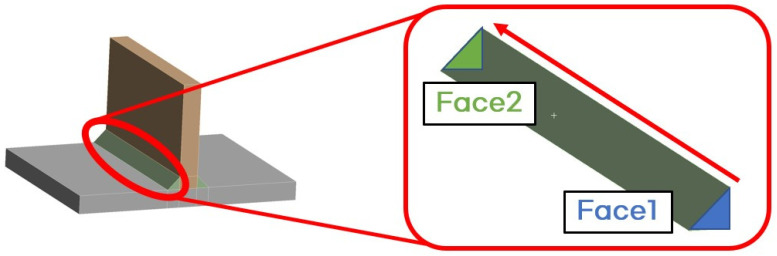
Details of setting welding direction.

This study implemented a simplified method to input welding temperature directly instead of specifying variables related to heat input, such as arc voltage and current. The welding temperature is typically set to exceed the material’s melting point. Additionally, as shown in Figure 1 and Figure 2, for the convenience of welding analysis, the welding direction and order can be easily configured. After modelling the welding bead and creating the mesh, users can select the starting and finishing face of the welding bead, and welding proceeds from the starting face to the finishing face. In other words, if the bead shape is provided, the weld path can be easily defined. Simultaneous welding analysis is also possible if multiple beads are assigned the same order priority.

Meanwhile, according to Totemeier & Tian [19], intercooling temperature also affects welding results. Therefore, in the toolkit developed for this study, an intercooling temperature can be set between welding passes, allowing for the desired cooling process between weld procedures.

In previous studies, Goldak’s double ellipsoidal model was used as a heat source [14,17,20,21,22,23]. To determine the dimensions of Goldak’s heat source model, the experiment should be conducted to measure the dimensions of the cross-section. However, if there is no data available from the experiment, it is recommended to apply the fore length of the heat source equal to half of the welding breadth, and the rear length of the heat source equal to twice the weld breadth [24]. Instead of using the method that applies Goldak’s double ellipsoidal model as in previous research, this study used the geometric characteristics of the weld bead to define the heat input region by specifying the fore length and rear length of the heat source. Following the recommendations of Goldak et al. [24], the fore length is set to half the width of the weld pool, and the rear length is set to twice the width of the weld pool. In this case, the fore length, rear length, and bead width define the heat input region, and the heat source moves along the bead according to the input welding speed. Additionally, the various welding conditions mentioned earlier can be adjusted, allowing for parametric studies. In this study, decoupled thermal elastic-plastic analysis was conducted using full 3D solid elements, with the thermal analysis being followed by the structural analysis. The Element Birth and Death (EBD) method was used to simulate the generation of the welding bead over time. The structural analysis following the thermal analysis utilised time-dependent thermal loads and boundary conditions. The results of the welding analysis included the six components of residual stress ($\sigma_{xx},\;\sigma_{yy},\;\sigma_{zz},\;\sigma_{xy},\;\sigma_{yz},\;\sigma_{zx}$) and the deformations in three directions ($u_{x},\;u_{y},\;u_{z}$). After the welding analysis, the initial residual stress and initial deformation of the T-bar were implemented using these results. Then, we were able to predict the ultimate compressive strength by conducting Non-linear finite element analysis (NLFEA).

## 3. Finite Element Model

### 3.1. T-Joint Fillet Welding

#### 3.1.1. Geometry for T-Joint Fillet Welding

**Figure 3:**
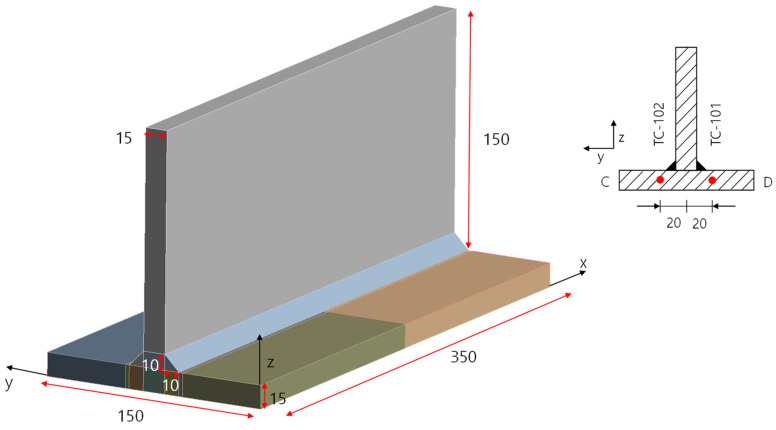
The geometry of the T-joint fillet welding model.

Since no welding experiments were conducted in this study, the geometry from a previous study by Perić et al. [6] was used for comparison with existing experimental results. As shown in Figure 3, there are two beads, each with a welding length of 350 mm. The plate and stiffener thickness is 15 mm, and the bead leg length is 10 mm. At the centre cross-section of the model, points TC-101 and TC-102 are located 20 mm to the left and right, respectively, from the midpoint of the plate’s thickness. At these points, the temperature changes during the welding process were measured.

#### 3.1.2. Material Property for T-Joint Fillet Welding

**Figure 4:**
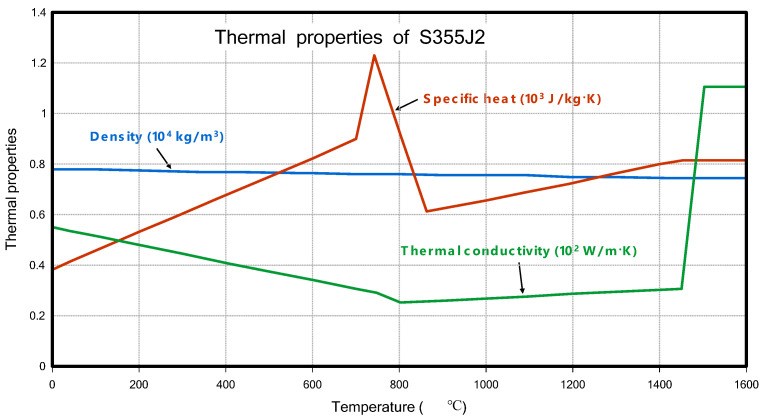
Thermal properties of S355J2.

**Figure 5:**
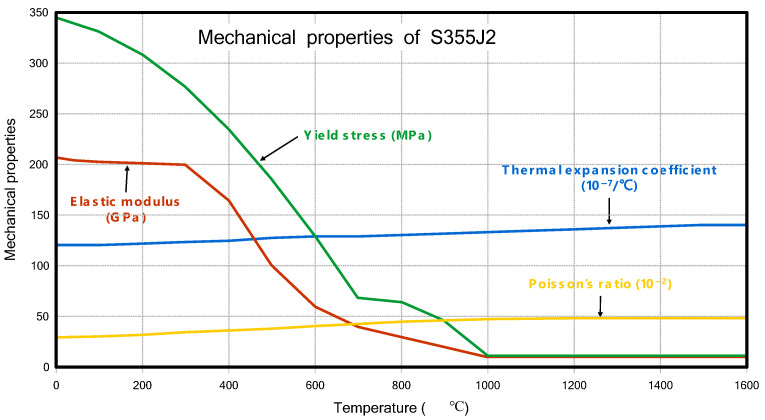
Mechanical properties of S355J2.

The material properties used in this study were also the same as those in the previous research [6], specifically the properties of S355J2. The thermal and mechanical properties are shown in Figure 4 and Figure 5, as shown below.

#### 3.1.3. Mesh and Boundary Conditions for T-Joint Fillet Welding

**Figure 6:**
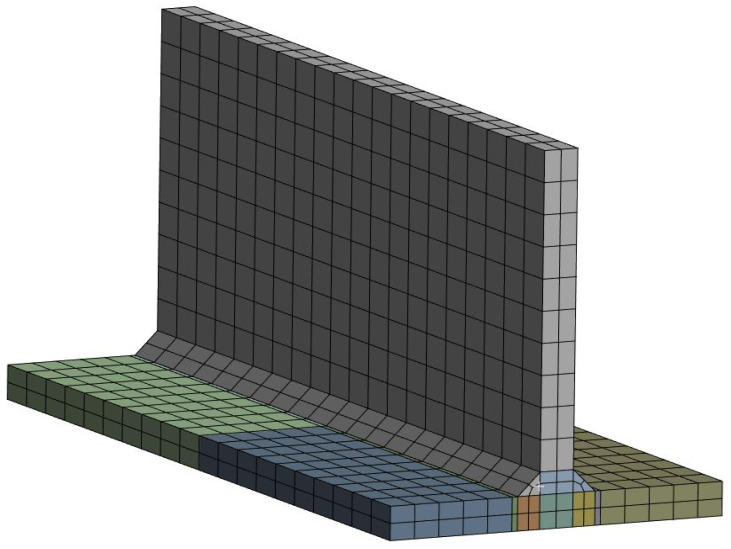
Mesh of the T-joint fillet welding model.

**Figure 7:**
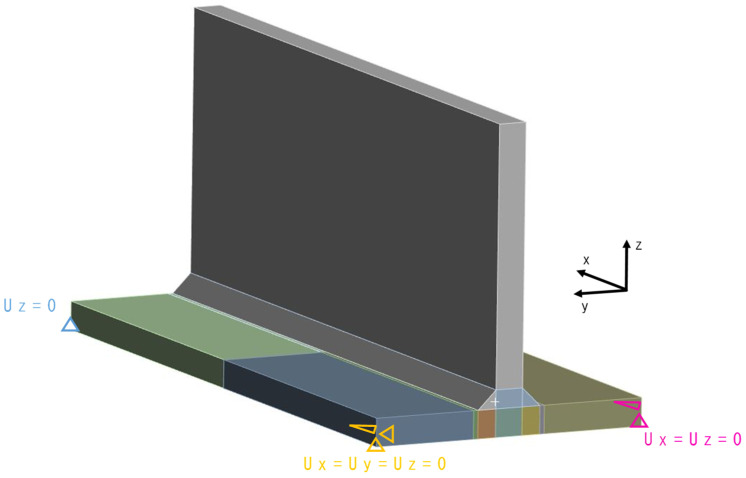
Boundary conditions of the T-joint fillet welding model.

The mesh of the analysis model is shown in Figure 6. A 3D solid, linear element was used, and to reduce the analysis time, the number of elements (NoE) is two in the thickness direction. The differently coloured parts in the figures represent distinct sub-parts. The boundary conditions from Perić et al. [6] were applied. The applied boundary conditions are shown in Figure 7. Boundary conditions were imposed at the middle plane in the thickness direction of the main plate. For the reference point, $U_{x}=U_{y}=U_{z}=0$ was applied; for the second point, $U_{x}=U_{z}=0$; for the third point, $U_{z}=0$; and for the remaining point, a free condition was applied.

**Figure 8:**
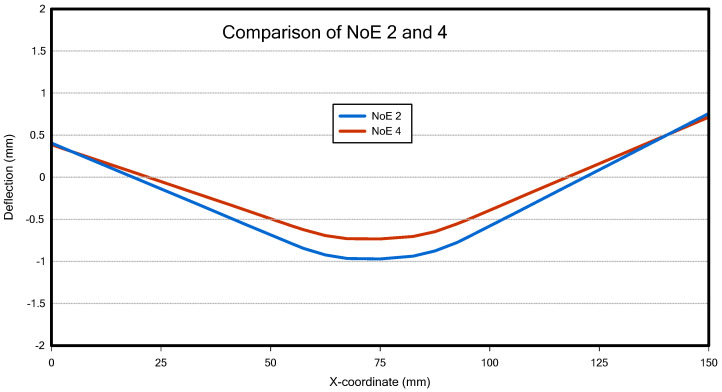
Deflection Comparison of NoE 2 and 4.

The number of elements (NoE) in the thickness direction must be an even number to apply boundary conditions to the central node in the thickness direction. Therefore, we performed the analysis with NoE set to 2 and 4 in the thickness direction and compared the z-deflection and computation time. As shown in Figure 8, there was no significant difference in z-deflection between NoE 2 and 4, while the computation time increased by a factor of 5. Furthermore, analysis time is also a critical factor for the parametric study aimed at obtaining thousands to tens of thousands of welding analysis data in the future. Hence, in this study, we conducted the analysis with NoE set to 2 in the thickness direction.

#### 3.1.4. Welding Conditions for T-Joint Fillet Welding

**Figure 9:**
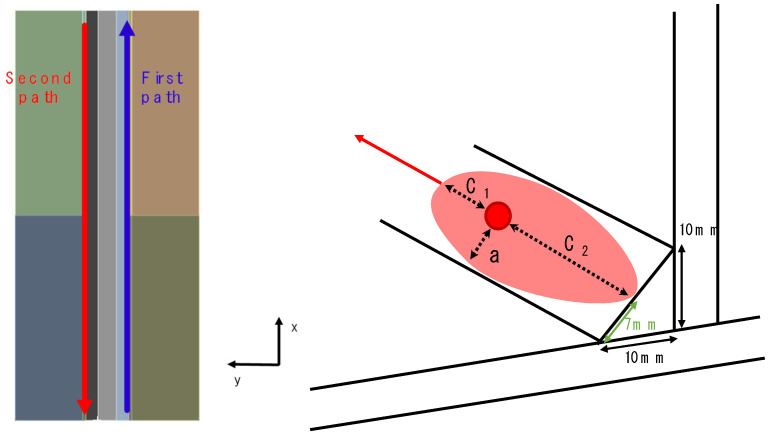
Welding path and geometry of simplified heat source.

First, the welding temperature was set to 1500 °C, which is the melting point of the material. The other welding conditions were wet according to the welding conditions in Perić et al. [6]. The welding speed was set to 404 mm/min (6.73 mm/s), the convection coefficient was set to 10 W/$m^{2}$°C, and the emissivity was set to 0.9. The welding direction is shown in Figure 8, where the welding directions of the first and second beads are different. Additionally, there is an intercooling period between the first and second bead welds, and the second bead welding begins after the temperature of the first bead drops to 200 °C. The simulation of the heat source was modelled using Goldak’s double ellipsoidal model. In the welding experiment and analysis by Perić et al. [6], the bead leg length was 10 mm. Geometrically, as shown in Figure 9, the half-width (a) of the bead surface is approximately 7 mm, with the fore length (C1) set to 7 mm, which is the half-width of the bead surface, and the rear length (C2) set to 28 mm, which is twice the bead surface width.

### 3.2. Butt Welding

#### 3.2.1. Geometry for Butt Welding

**Figure 10:**
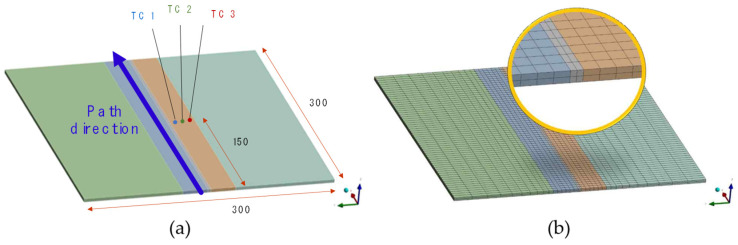
Geometrical features and mesh of the butt-welding model. (**a**) Geometry of the butt-welding model (**b**) Mesh of the butt-welding model.

Groove welding is a predominant form of welding employed in industry, wherein two components are joined by welding their mating surfaces. Based on the degree of penetration achieved at the joint, groove welding is categorised into complete (full penetration) welding and incomplete (partial penetration) welding, with specific industry guidelines applicable to each category. In the present study, we have conducted a welding analysis focusing on the most representative butt weld. To verify the accuracy of the current welding model, we conducted a comparison with the previous study [25]. The same model and mesh as those used in the reference study (see Figure 10) were adopted, with the material properties of St37 (low carbon steel) applied. To demonstrate good agreement with the experimental temperature data reported in their paper, we compared the experimental and FEM temperature data at three points located 10 mm, 20 mm, and 30 mm away from the bead centre, TC-1, 2, and 3, respectively.

#### 3.2.2. Material Property for Butt Welding

**Figure 11:**
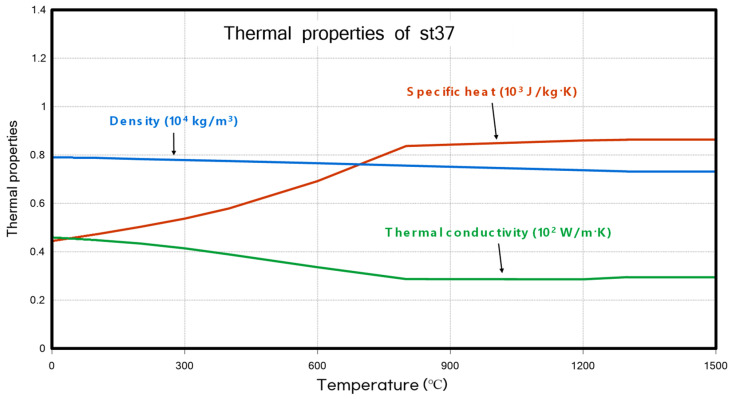
Thermal properties of st37.

**Figure 12:**
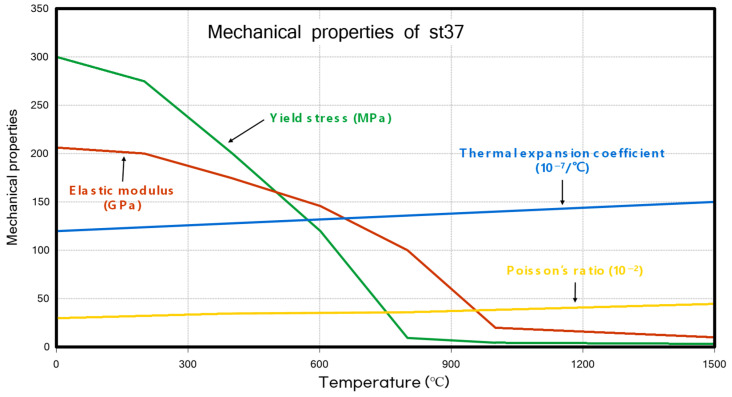
Mechanical properties of st37.

The material properties used in this study were the same as those in the previous research [25], St37. The detailed thermal and material properties are shown in Figure 11 and Figure 12, respectively.

#### 3.2.3. Mesh and Boundary Conditions for Butt Welding

The mesh size effect is one of the important considerations for the numerical simulation [26,27]. This study adopted the same mesh as those used in the reference study [25] (referred to Figure 10).

In the thickness directions, the number of elements is two. In the transverse direction, the central weld area was composed of a fine mesh, while the mesh became increasingly coarse as it extended further from the weld area towards the surrounding regions. In this study, only the top nodes of the four corners were constrained to all three directions of translation.

#### 3.2.4. Welding Conditions for Butt Welding

The welding temperature was set to 1500 °C, which is the melting point of the material, as in the case of T-joint fillet welding, and the other welding conditions were applied in accordance with the conditions from previous research [25]. Welding speed is 0.5 m/min (8.33 mm/s). The bead width was set to 4 mm, which is the same as the thickness of the plate. Thus, geometrically, we set the fore length of the heat source to 2 mm, and rear length of the heat source to 8mm. The convection coefficient and emissivity were set to 30 W/$m^{2}$°C, 0.9, respectively, to all surfaces of the plate.

## 4. Analysis Results

### 4.1. Thermal Analysis

#### 4.1.1. Thermal Analysis Results of T-Joint Fillet Welding

As previously described, a transient thermal analysis was performed to simulate the welding process over time using the EBD (Element Birth and Death) method. After welding the first bead and intercooling, the second bead was welded in the opposite direction. After the second bead welding, the model was cooled down to room temperature (25 °C).

**Figure 13:**
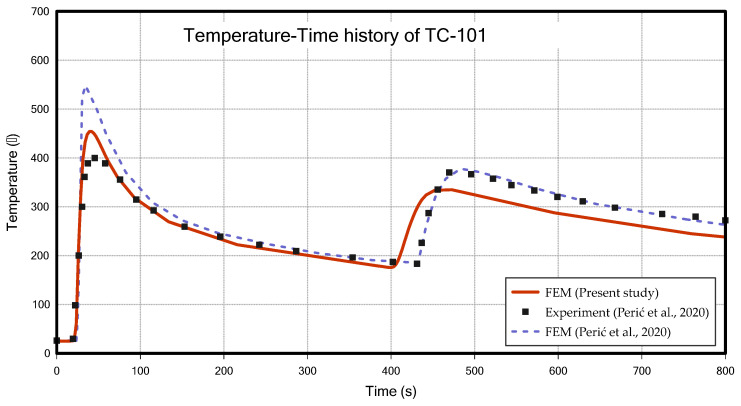
Temperature–time history of TC-101 (T-joint fillet welding) [6].

**Figure 14:**
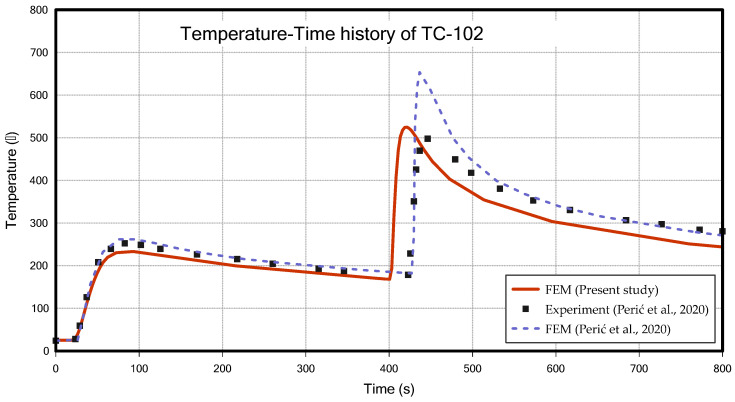
Temperature–time history of TC-102 (T-joint fillet welding) [6].

**Figure 15:**
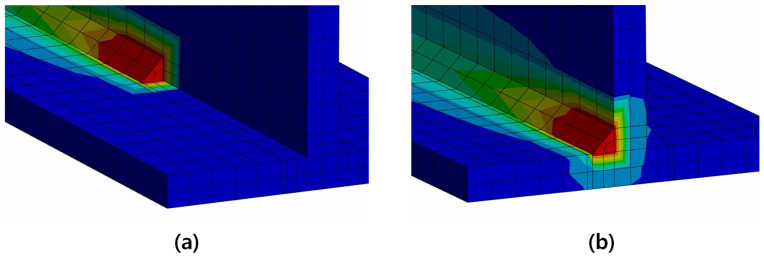
Thermal analysis results of the typical cases: (**a**) thermal analysis at 31 s, (**b**) thermal analysis at 431 s.

Figure 13 and Figure 14 show the temperature changes over time measured at the TC-101 and TC-102, respectively, comparing the results of this study with the result from Perić et al. [6]. Although the peak values showed a maximum difference of about 11% in TC-101, the overall temperature profile was very similar between the two studies, and the temperature profile of TC-102 is more similar to the experiment data of Perić et al. [6] than FEM result of Perić et al. [6]. The two results showed a slight difference in the timing of the second bead welding. In previous research [6], the intercooling was applied based on time (352 s), whereas in this study, it was applied based on a temperature condition (200 °C). In this study, the intercooling temperature was adjusted appropriately to align the second bead welding start time as closely as possible with the study by Perić et al. [6]. Figure 15a,b show the configurations (31 s, 431 s) during the thermal analysis, respectively.

#### 4.1.2. Thermal Analysis Results of Butt Welding

**Figure 16:**
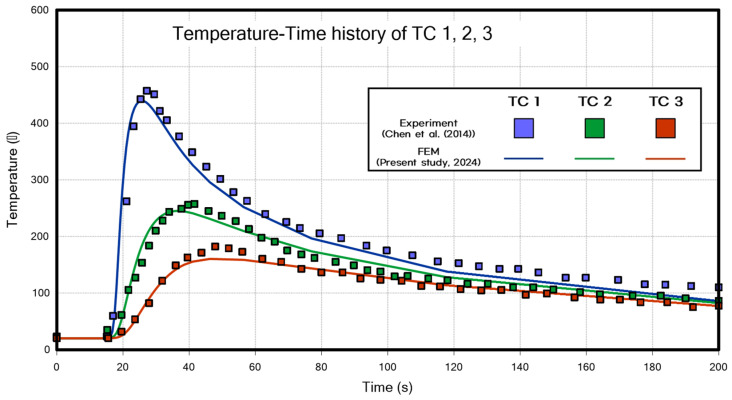
Temperature–time history of TC 1, 2, 3 (Butt welding) [25].

Figure 16 shows the temperature measurement points and the comparative results of the temperature data at these locations. Although there are discrepancies in the peak values of the analysis results, the overall trends exhibit good agreement. To validate the structural analysis, the experimental data from the referenced paper [25] were compared with the FEM analysis results obtained in this study.

### 4.2. Structural Analysis

#### 4.2.1. Structural Analysis Results of T-Joint Fillet Welding

**Figure 17:**
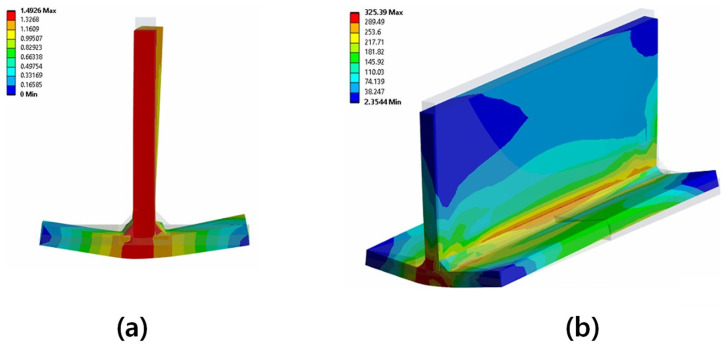
Deformation (**a**) and residual stress distributions (**b**) (T-joint fillet welding).

The key results of the welding analysis are welding-induced deformations and residual stresses. First, examining the deformations caused by welding, longitudinal shrinkage, transverse shrinkage, and angular distortion were observed. It is challenging to compare deformations due to the difference in the boundary conditions. Therefore, in this study, the only comparison of deformation was made with the result of Perić et al. [6]. Figure 17a shows the deformations, and Figure 17b displays the residual stress distributions. These results align well with the commonly known effects of welding.

**Figure 18:**
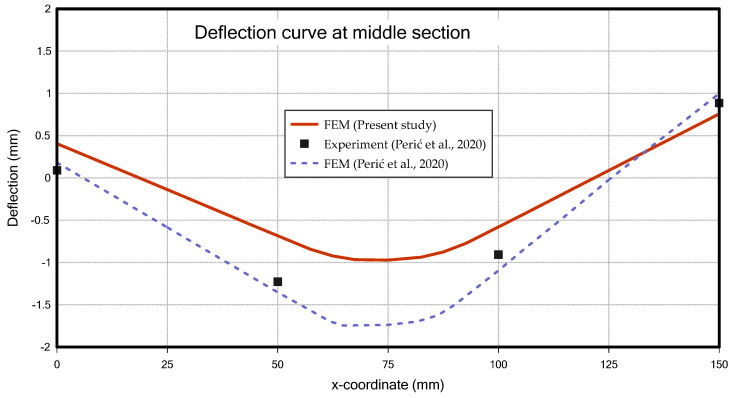
Deflection curve at middle section (T-joint fillet welding) [6].

Figure 18 compares the results of this study with those of Perić et al. [6], showing the z-deflection in the middle section. In comparison with the experimental data, the result from this study showed a slightly larger error than the FEM results of Perić et al. [6]. This discrepancy is likely due to the fact that the welding temperature was set to the material’s melting point in this study, which is lower than the actual welding temperature, thereby reducing the overall heat input.

**Figure 19:**
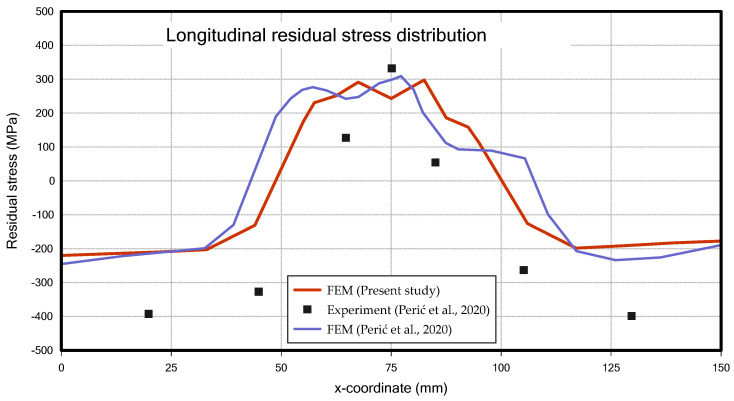
Longitudinal residual stress distribution at middle section (T-joint fillet welding) [6].

In the case of residual stress, when looking at the longitudinal residual stress, as shown in Figure 19, tensile residual stress occurred in the centre of the member, where heat input was applied by the bead, and compressive residual stress was generated in the surrounding areas of the member. This distribution is similar to the findings of Perić et al. [6] and is consistent with the research results of Khan & Zhang [28] and Paik & Sohn [29], who studied residual stresses caused by welding.

The residual stress in this study shows a more symmetrical distribution compared to the results of Perić et al. [6]. However, the maximum residual stress is slightly lower than experimental data from previous research [6], which can be attributed to the fact that the welding temperature was set to the melting point of the material, which is lower than the actual welding temperature, resulting in lower overall heat input.

#### 4.2.2. Structural Analysis Results of Butt Welding

**Figure 20:**
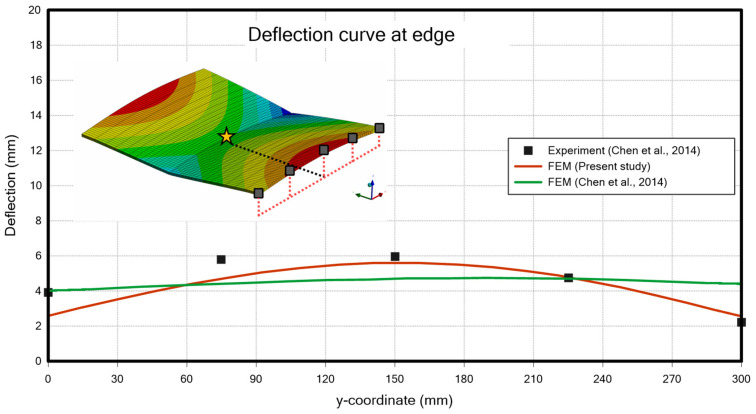
Deflection curve at edge (butt-welding) [25].

Since the methodology for groove welding has been validated, it is applied to butt welding with an arc shape to verify the convergence of the analysis. During the analysis, boundary conditions were applied by assigning fixed supports to the corner nodes on the top surface of the welding plate. The welding bead was designed to trace an arc shape centred on the midpoint of the root gap. The deformation of the plate for the given model is shown in Figure 20. The results align well with the well-established outcomes for typical butt welds.

### 4.3. Welding-Induced Residual Stress and Deformation as Initial Deflection

**Figure 21:**
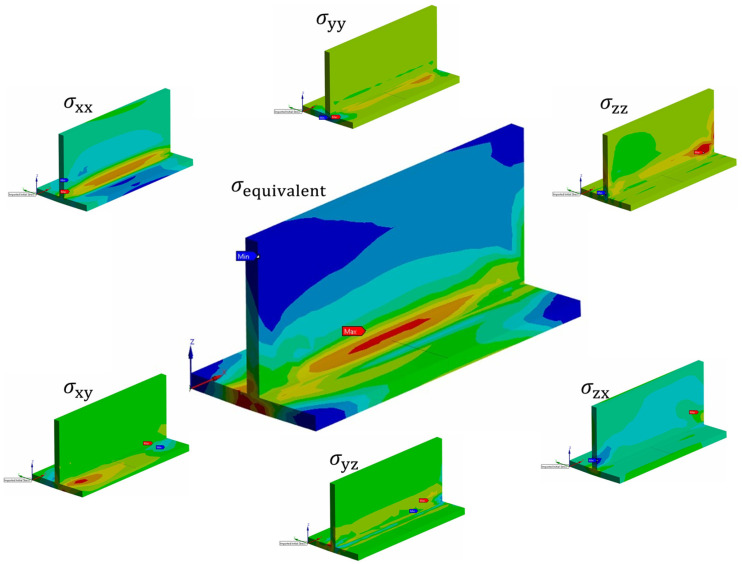
Equivalent stress and six stress components.

As mentioned earlier, the main results of the welding analysis in this study are the residual stress and deformation caused by welding. The six stress components ($\sigma_{xx},\;\sigma_{yy},\;\sigma_{zz},\;\sigma_{xy},\;\sigma_{yz},\;\sigma_{zx}$) at each node can be extracted to assess the residual stress of the member (Figure 21). Various subsequent studies, such as compression and fatigue analysis, can be conducted using the deformed shape and residual stress from the welding analysis as the initial deflection.

**Figure 22:**
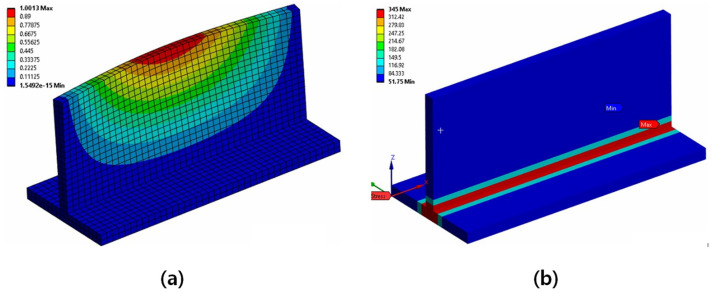
Analysis results for the typical cases: (**a**) Eigenmode buckling shape, (**b**) Idealised residual stress.

This study conducted a compression analysis after the T-joint fillet welding analysis for demonstration. For comparison, a traditional analysis method was performed as a comparative analysis. In this case, for initial deformation, the buckling shape mode from eigenmode analysis was applied, and the applied initial deflection is average-level, as shown in Equation (1) defined by Smith et al. [30], with plate slenderness ratio (β) of 0.4084. The results of the eigenmode analysis are shown in Figure 22a. For the residual stress, an idealised residual stress formula for plates was used to input the values. The residual stress method follows the previous research approach [29], specifically applying the average level values. The results from the applied methods are shown in Figure 22b.

(1) $$\frac{w_{opl}}{\beta^{2}t}=\left\{\begin{array}{c} 0.025\;\;\;for\;a\;slight\;level\\ 0.1\;\;\;for\;a\;average\;level\\ 0.3\;\;\;for\;a\;severe\;level \end{array}\right.$$

**Figure 23:**
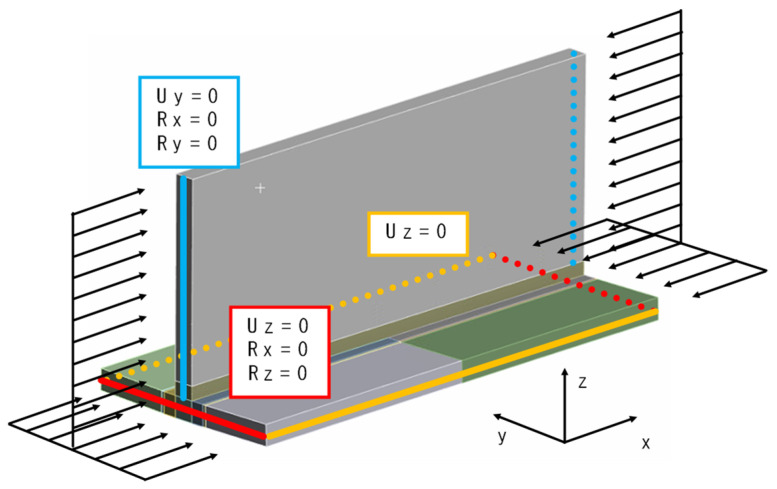
Boundary conditions and load conditions of compression analysis.

Subsequently, using the same mesh and material properties as before, the boundary conditions and loading conditions are slightly changed. A simply supported boundary condition was applied. It is known that the simply supported boundary condition is widely adopted in predicting the ultimate strength of the unstiffened [31,32,33,34] and stiffened panels [35,36,37,38,39] subjected to compressive loads. For the edges parallel to the y-axis (4 plate edges), displacement in the z-direction and rotations around the x-axis and z-axis were constrained. For the edges parallel to the z-axis (2 web edges), displacement in the y-direction and rotations around the x-axis and y-axis were constrained, as shown in Figure 23.

There were three nodes in the thickness direction of the plate. When setting up boundary conditions, only the middle node at each edge was selected to fully apply the rotational effects without imposing constraints on the other nodes. For the loading conditions, all nodes in the thickness direction were selected to ensure uniform compression. Compression was applied using displacement control, with a compression of 10% of the member’s length along the x-axis. To apply the compression symmetrically, 5% displacement was applied to each of the two opposing end surfaces.

**Figure 24:**
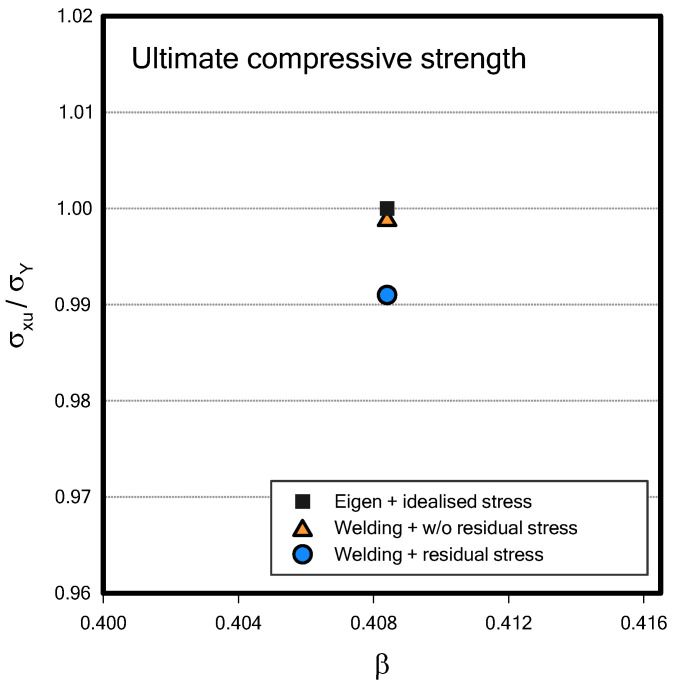
Ultimate compressive strength among three analysis cases.

Due to the low plate slenderness ratio (β), there was no significant difference among the three cases, as shown in Figure 24. Despite the lowness of plate slenderness ratio (β), conventional analysis (eigen + idealised residual stress) assesses the strength most highly, followed by the analysis case of only considering welding-induced deformation. When considering both welding-induced deformation and residual stress, the ultimate compressive strength of the component is the lowest among the three cases (about 1% less than eigen + idealised residual stress). However, the present study only considered a limited number of the scenarios. Therefore, additional studies, i.e., ULS comparison, effect of residual stress, and modelling technique, are recommended to be conducted in the future as widely investigated by Li et al. [39] using 2D shell element.

## 5. Conclusions

In this study, finite element analysis was utilised to perform welding simulations to increase the convenience of welding analysis and facilitate the analysis of more complex models. The welding analysis was divided into thermal and structural analyses. During the thermal analysis, the element birth and death (EBD) method was used to simulate the welding process, while the structural analysis was performed using time-dependent thermal loads and boundary conditions. Subsequently, a compression analysis was performed using the welding-induced deformation and residual stress distribution as initial imperfections. The ultimate compressive strength was then analysed by comparing the results with those obtained from the conventional compression analysis method. The main results of this study are as follows:

- An ACT (Analysis Customization Toolkit) was developed using the commercial FEA program ANSYS to simplify welding analysis. The key features of the ACT include simplified weld path designation, simultaneous and sequential welding functionality, an intercooling function, and a simplified heat source model using the bead’s geometrical features. These features are expected to be advantageous for welding analysis of more complex structures.
- Although the convenience of welding analysis was enhanced, a full 3D solid element thermal elastic-plastic welding analysis was performed to maintain the accuracy of the results. Each analysis took less than 10 min, and the results were verified to be similar to those from previous studies and experiments.
- The ACT-based welding analysis method was applied to two commonly used welding types: T-joint fillet welding and butt welding. The analysis yielded satisfactory results, and these welding methods are expected to be applied to complex and large structures for welding analysis.
- A compression analysis was performed using the deformation and residual stress distribution obtained from the welding analysis as initial deflections. Moreover, the most conservative result is obtained compared with conventional analysis.

In this study, acceptable results were obtained despite simplifying the analysis conditions to improve the convenience of welding analysis. To achieve more accurate analysis, further research will be required to accurately determine the precise temperature of the weld bead within real welding environments. In addition, a wide range of the stiffened panel’s geometry and material properties shall be further considered to investigate the ultimate compressive strength characteristic by revising the plate slenderness and column slenderness ratios. Lastly, the effect of the initial deflection shape should also be further investigated as highlighted by Wang et al. [40]. The ACT developed in this study is expected to be useful for various welding-related research in the future.

## Data Availability

Data available on request.
